# Letter to the Editor – unraveling molecular characteristics and functional exploration of panoptosis for prognosis stratification in lung adenocarcinoma: a tumor marker prognostic study

**DOI:** 10.1097/JS9.0000000000003428

**Published:** 2025-09-05

**Authors:** Man Sun, Dan Zang, Jun Chen

**Affiliations:** Department of Oncology, The Second Hospital of Dalian Medical University, Dalian, Liaoning, China

*To the Editor,*

We read with great interest the recent article by Hu *et al*^[1]^, which proposed and validated a PANoptosis-based prognostic model (PANscore) for lung adenocarcinoma using transcriptomic and experimental data. With increasing interest in integrating regulated cell death programs into prognostic modeling frameworks, PANoptosis represents a novel but mechanistically elusive axis under active investigation. Such models demand rigorous validation across mechanistic, statistical, and clinical axes to realize translational value. This study complies with the TITAN 2025 guidelines for transparent and ethical reporting of AI-assisted research^[2]^.

First, while PANoptosis has been proposed as a unified inflammatory cell death pathway integrating pyroptosis, apoptosis, and necroptosis, its mechanistic identity remains loosely defined. Hu *et al* developed the PANscore from 66 literature-curated genes, yet without confirming their specificity to PANoptosis. Transcriptomic patterns and cleavage of shared effectors such as CASP3, GSDMD, and MLKL do not establish activation of the PANoptosome – the central executor of PANoptosis^[3]^. As such, PANscore may reflect general cell death rather than a distinct PANoptotic program^[4]^. Mechanistically labeled models demand functional validation, including disruption or reconstruction of core PANoptosome components, to ensure conceptual precision and clinical relevance.

Second, although the authors explored 101 machine learning combinations, the model’s statistical foundation remains vulnerable. Feature selection was based solely on univariate Cox regression without multiple testing correction, risking false-positive inclusion. In a high-dimensional context, the lack of penalized regression methods and nested cross-validation raises concerns of overfitting and inflated performance^[5]^. Validation cohorts, drawn exclusively from similar public datasets, offer limited demographic and clinical diversity^[6]^. Without internal robustness checks or prospective external datasets, the model falls short of the statistical rigor essential for clinical prognostic tools. In precision oncology, methodological discipline is not optional – it defines translational credibility.

Third, although the study incorporates bulk and single-cell transcriptomics, pharmacogenomics, immunotherapy cohorts, and experimental assays, these data layers remain fragmented rather than mechanistically integrated. Key components such as YWHAG are not functionally mapped to PANscore outputs, and associations with treatment response remain purely correlative^[7]^. No unified model connects PANoptosis activity to therapeutic outcomes, limiting biological interpretability. In translational oncology, multi-omic frameworks must move beyond linear stacking toward mechanistic convergence. Recent integrative strategies – such as OmniFuse-type architectures – highlight the necessity of latent factor alignment to enable cross-modal causal inference and therapeutic translation^[8]^. The absence of such strategies in this study undermines the explanatory coherence and translational clarity of the proposed model.

Finally, although PANscore is positioned as a clinically actionable biomarker, its diagnostic readiness remains unproven. Developed solely from research-grade RNA sequencing data, the model has not been evaluated on clinically deployable platforms such as quantitative PCR, NanoString, or targeted gene panels – modalities essential for routine implementation^[9]^. No expression thresholds, minimal gene sets, or assay standardization strategies – such as compatibility with FFPE tissue, applicability to biopsy samples, or inter-laboratory reproducibility – were reported^[10]^. Furthermore, all validations were confined to retrospective, demographically limited public cohorts, without prospective or multicenter evidence. In modern oncology, clinical translation requires not only prognostic validity but also logistical feasibility, technical scalability, and regulatory alignment. Without a defined implementation path, PANscore risks remaining a computational construct with limited clinical utility.

In summary, while Hu *et al* offer a timely effort to integrate PANoptosis into prognostic modeling for lung adenocarcinoma, the proposed framework falls short in mechanistic specificity, statistical rigor, and translational readiness. As precision oncology shifts from associative insights to clinically actionable tools, future models must be grounded in validated biology, compatible with diagnostic platforms, and aligned with regulatory pathways. Embedding such signatures into standardized, demographically inclusive pipelines – while harmonizing with evolving diagnostic standards, quality metrics, and health technology assessment frameworks – will be essential to ensure predictive validity and system-level adoption. Benchmarking against initiatives such as ESCAT and NICE HTA may further support their co-development alongside targeted therapies. This correspondence seeks to advance the translational refinement and health system integration of PANoptosis-based biomarkers, ultimately promoting their evolution from conceptual models to clinically deployable tools in surgical oncology.

## Data Availability

None.
